# The human Ago2 MC region does not contain an eIF4E-like mRNA cap binding motif

**DOI:** 10.1186/1745-6150-4-2

**Published:** 2009-01-21

**Authors:** Lisa N Kinch, Nick V Grishin

**Affiliations:** 1 Howard Hughes Medical Institute and Department of Biochemistry, University of Texas Southwestern Medical Center, 5323 Harry Hines Blvd, Dallas, TX 75390-9050, USA

## Abstract

**Background:**

Argonaute (Ago) proteins interact with small regulatory RNAs to mediate gene regulatory pathways. A recent report by Kiriakidou et al. [1] describes an MC sequence region identified in Ago2 that displays similarity to the cap-binding motif in translation initiation factor 4E (eIF4E). In a cap-bound eIF4E structure, two important aromatic residues of the motif stack on either side of a 7-methylguanosine 5'-triphosphate (m^7^Gppp) base. The corresponding Ago2 aromatic residues (F450 and F505) were hypothesized to perform the same cap-binding function. However, the detected similarity between the MC sequence and the eIF4E cap-binding motif was questionable.

**Results:**

A number of sequence-based and structure-based bioinformatics methods reveal the reported similarity between the Ago2 MC sequence region and the eIF4E cap-binding motif to be spurious. Alternatively, the MC sequence region is confidently assigned to the N-terminus of the Ago piwi module, within the mid domain of experimentally determined prokaryotic Ago structures. Confident mapping of the Ago2 MC sequence region to the piwi mid domain results in a homology-based structure model that positions the identified aromatic residues over 20 Å apart, with one of the aromatic side chains (F450) contributing instead to the hydrophobic core of the domain.

**Conclusion:**

Correct functional prediction based on weak sequence similarity requires substantial evolutionary and structural support. The evolutionary context of the Ago mid domain suggested by multiple sequence alignment is limited to a conserved hydrophobicity profile required for the fold and a motif following the MC region that binds guide RNA. Mapping of the MC sequence to the mid domain structure reveals Ago2 aromatics that are incompatible with eIF4E-like mRNA cap-binding, yet display some limited local structure similarities that cause the chance sequence match to eIF4E.

**Reviewers:**

This article was reviewed by Arcady Mushegian, Chris Ponting, and Igor Jouline (nominated by Igor Zhulin).

## Background

Argonaute (Ago) proteins interact with small regulatory RNAs to guide RNA silencing mechanisms and to regulate translation. Members of the Ago family possess two conserved regions identified by sequence analysis: an N-terminal PAZ (named for Piwi-Argonaute-Zwille) domain followed by a C-terminal piwi domain [2]. Structural studies of Ago proteins have shed light on the molecular function of these domains (for a review see [3]). The PAZ domain adopts an oligonucleotide/oligosaccharide-binding fold (OB-fold)[4,5] that mediates 3' end recognition of guide RNA[6,7], while piwi includes a C-terminal Ribonuclease H (RnaseH)-like fold[8,9] that catalyzes guided cleavage of mRNA[10]. A second subdomain of piwi, designated as the 'mid' domain, is located between the N-terminal PAZ domain and the catalytic RnaseH-like domain. The mid domain, which binds the 5' end of guide RNA, represents an integral structural and functional unit of the defined piwi region, and is often referred together with the catalytic domain as a single piwi module[3].

Kiriakidou et al. describe a motif (MC) within the mid domain of Ago proteins that bears some resemblance to a translation initiation factor eIF4E sequence motif responsible for binding the m^7^G cap of mRNA[1]. The authors identify two aromatic residues within the MC region that are conserved between eIF4E (W56 and W102) and human Ago2 (F470 and F505) and suggest an analogous cap-binding function for the identified residues. A cocrystal structure of eIF4E bound to m^7^Gppp [PDB: 1l8b] illustrates the cap binding mode [11]. The m^7^Gppp moiety stacks between the two identified tryptophan aromatic side chains (W56 and W102), forming hydrogen bonds with the side chain of a neighboring glutamine residue (E103), and a van der Waals interaction with the side chain of an additional tryptophan (W166). Additional conserved eIF4E residues (W102, W166, R112, K162, and R157) form salt bridges and hydrogen bonds with the cap analog phosphates. Despite a rather limited preservation of cap binding residues between the identified MC motif and the eIF4E sequence, the authors pursued establishing a cap-binding function for Ago2.

Based on sequence analysis of the Ago protein family, we confirm previously described homology [2,3,12] between metazoan Ago sequences (such as *hs *Ago2) and prokaryotic piwi structures. We present a multiple sequence alignment between the corresponding mid domains of eukaryotic Ago representatives and prokaryotic piwi structures that allows confident mapping of key Ago2 residue positions. A resulting homology-based structure model of the Ago2 mid domain illustrates the spatial arrangement of both the identified aromatic residues in the described MC motif. When compared to the experimentally determined m^7^G cap bound eIF4e structure, the positions of the Ago2 aromatic residues are inconsistent with analogous cap-binding modes. Such a result brings into question both the assignment of the MC sequence region as a cap-binding motif, and the specific participation of the two identified aromatic residues from Ago2 in forming base-stacking interactions with cap.

## Results and discussion

### All piwi homologs include the MC sequence region

The piwi sequence region was initially defined based on sequence similarity[2], and subsequently determined structures revealed piwi to include an N-terminal mid domain followed by a C-terminal catalytic domain[8,9]. Sequence limited to the mid domain structure [PDB: 1yvu] confidently identifies a number of Ago homologs (results for representatives summarized in Table 1). Identified sequences encompass all elements of the mid domain fold, including the MC sequence region (Table 1, Final Coverage). Thus, Ago sequences described by Kiriakidou et al. as lacking the MC region [1] (plants, archaea, fission yeast, *D. melanogaster *Ago2, extended *C. elegans *family members, and piwi-like sequences) do include structural elements of the MC motif, which represents an integral part of the overall fold.

**Table 1:** Identification of representative Ago mid domain sequences

Hit	GI	Round	E-value	Range	Initial Coverage	Final Coverage
*Hs *Piwi4	116242716	4	3.0E-05	515–605	0.67	0.90
*Sp *Ago	19075282	5	1.0E-05	446–558	0.83	0.99
*At *Zll	15239989	5	3.0E-04	566–682	0.86	0.99
*Dm *Ago2	24664664	5	4.0E-03	868–943	0.56	0.96
*Dm *Ago1	24653501	6	6.0E-22	587–698	0.82	0.93
*Hs *Ago2	29171734	6	4.0E-20	461–574	0.84	0.93
*Ce *Alg1	25148113	6	5.0E-20	596–717	0.90	0.96
*Ce *Ppw2	17510939	7	3.0E-06	552–665	0.84	0.96

PSI-BLAST results reported for initial detection of representative hits using a sequence query corresponding to the mid domain structure. Coverage is calculated as the quotient of the range of either the initial hit or the hit after convergence and the range of query.

**Figure 1:**
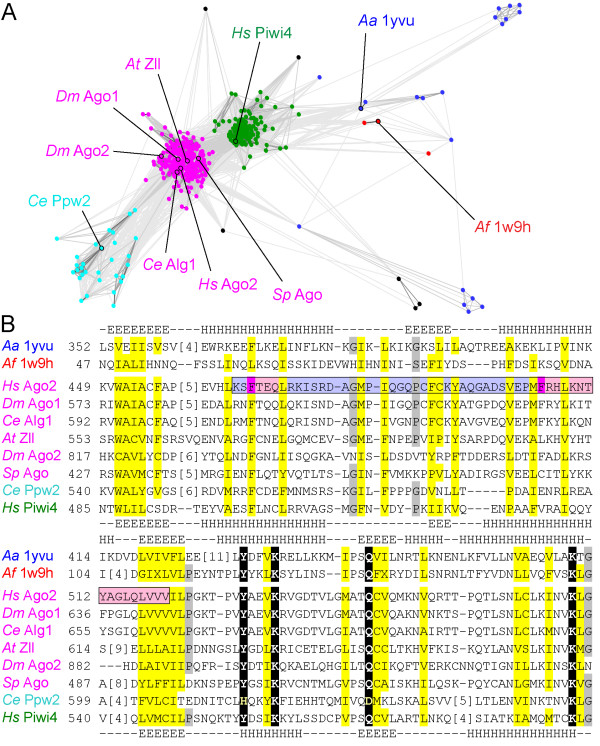
**Similarity plot and alignment of mid domain sequences**. (A) A two-dimensional cluster graph groups mid domain sequences. Nodes represent sequences connected by grayscale edges (high to low similarity colored black to gray). Three groups include Ago sequences (magenta), piwi-like sequences (green), and nematode paralogs (cyan). Sparsely connected nodes are colored by superkingdom: eukaryotes (black), bacteria (blue), and archaea (red). Representatives are labeled with species abbreviation and protein name or PDB id. Species abbreviations: *Aa, A. aeolicus; Af, A. fulgidus; At, A. thaliania; Ce, C. elegans; Dm, D. melanogaster; Hs, H. sapiens*; and *Sp, S. pombe*. (B) Ago/piwi multiple sequence alignment highlights conservations. Sequences are labeled and colored as in (A). Residue positions are colored by Ago conservation: hydrophobic (yellow), small (grey), and conserved polar (black). Residue numbers are to the left of the alignment, with omitted residues in brackets. Observed secondary structures (E strand and H helix) are above the alignment, while predicted secondary structures are below. Ago2 MC is boxed, with identified aromatics highlighted magenta, locally similar secondary structures (to eIF4E) highlighted pink, and dissimilar regions (to eIF4E) highlighted light blue.

Similar to previous evolutionary analysis of piwi proteins [13-15], identified mid domain sequences form three distinct groups that include Ago sequences, piwi-like sequences, and a set of nematode paralogs (Figure 1A). Identified prokaryotic homologs display more sparse overall connections, diverging from the three main eukaryotic groups. To help align the divergent sequences, an Ago2 mid domain sequence was queried against a database of structures using two different sensitive homology detection methods (COMPASS[16] and HHpred[17]). The sensitivity of these methods derives from comparing a multiple sequence alignment profile built from query homologs to similarly derived profiles of existing structures. Human Ago2 identified piwi structure representatives as the top hits (Table 2). The resulting alignments allowed confident mapping of the human Ago2 sequence to the mid domain structures (Figure 1B). Secondary structure predictions for the Ago family match those observed in the structures. Position specific Ago family conservations revealed in a multiple sequence alignment define a hydrophobicity profile for the fold. Similar conservations extend to sequences from the two other groups (represented by Hs Piwi4 and Ce Ppw2) as well as to the more distant prokaryotic sequences of the structures, supporting the final multiple sequence alignment.

**Table 2:** Identification of structures corresponding to Ago2 mid domain

Hit	PDBid	Score	E-value
COMPASS Results			
*A. aeolicus *Argonaute	2F8S_A	267	1.50E-28
*A. fulgidus *Piwi domain	1W9H_A	264	7.96E-26
*P. furiosus *Argonaute	1U04_A	221	1.87E-15
HHPRED Results			
*A. aeolicus *Argonaute	1yvu_A	126.2	7.60E-20
*P. furiosus *Argonaute	1u04_A	104.4	7.90E-16
*A. fulgidus *Piwi domain	1w9h_A	88.1	8.30E-13

Top-ranked structure hits with significant scores to Ago2 mid domain sequence query using COMPASS or HHPRED sequence-based detection methods.

Despite identifying a diverse group of Ago and piwi-like sequence homologs, no sequence representing eIF4E was detected using exhaustive PSI-BLAST searches against the non-redundant sequence database. In an attempt to recreate the link reported in Kiriakidou et al. [1], a single eIF4E-1 sequence from *C. elegans *[SwissProt: O45551] was identified in the first round of PSI-BLAST by querying the Ago2 mid domain against the limited sequence database used by the authors (SwissProt). However, the sequence was detected with a below threshold confidence score (E-value 0.17), and upon subsequent PSI-BLAST iterations, the sequence becomes undetectable (E-value greater than 100). Both the mid domain sequence from a close Ago2 homolog (*Dm *Ago1) and the corresponding MC sequence from the eIF4E structure [PDB: 1l8b] find the closest respective sequences of the other family with much worse confidence (E-value 15 and 77, respectively). Such results suggest the MC sequence region alignment to eIF4E represents a spurious PSI-BLAST hit between sequences displaying chance similarity.

### Ago2 structure model reveals incompatible eIF4E-type cap binding

**Figure 2:**
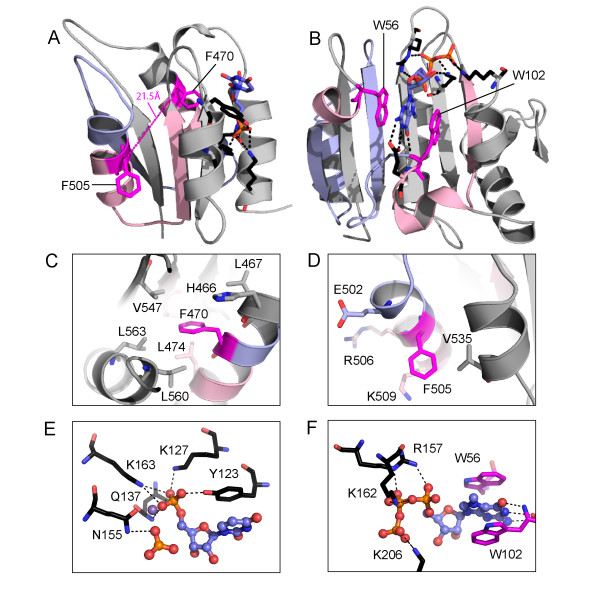
**Comparison of Ago2 structure model to EIF4e cap-binding mode**. (A) A ribbon diagram depicts the Ago2 mid domain homology-based structure model (2bgg template). The distance between labeled aromatic residue (magenta stick) C-alpha carbons is indicated. Locally similar structure elements (to eIF4E) surrounding these residues are colored pink, with the remaining MC region colored light blue. Conserved residues (black stick) form hydrogen bonds (dashed lines) to the 5'phosphate of guide RNA (slate). (B) A ribbon diagram illustrates the eIF4E cap-binding mode (1l8b). M^7^G stacks between labeled aromatic residues (magenta stick) and forms hydrogen bonds to additional conserved residues (black stick). Cap-binding motif corresponding to MC region is colored and depicted as in (A). (C) and (D) Ago2 aromatic residue surroundings (1w9h template) are zoomed in around the C-alpha position of F450 (C) or F505 (D). Neighboring residues are rendered as sticks, colored as above, and labeled with corresponding Ago2 numbering. (E) A zoom of the piwi (2bgg) guide RNA 5'nucleotide-binding pocket. Conserved residues (black stick) that hydrogen bond (dashed lines) to nucleotide (ball and stick) phosphates or stack with the 5' nucleotide base are labeled according to the corresponding Ago2 residues. A divalent cation (purple sphere) is found between the 5' phosphate and a phosphate from the third guide RNA nucleotide. (F) A zoom of the eIF4e cap-binding pocket (1l8b) with the m^7^Gppp cap analog oriented similarly to the piwi 5'guide RNA nucleotide to illustrate similarities. Conserved eIF4e residues (black stick) that hydrogen bond to nucleotide phosphates and stack with the nucleotide base are labeled.

The Piwi mid domain multiple sequence alignment allows building and evaluation of homology-based structure models for Ago2. Input alignments of the Ago2 mid domain with several structure templates corresponding to the *A. fulgidus, A. aeolicus, and P. furiosus *sequences produced similar models. Since the *A. fulgidus *templates represent better resolution structures, the final illustrated Ago2 models are based on the mid domain from *A. fulgidus *Piwi alone [PDB: 1w9h] and bound to double-stranded guide [PDB: 2bgg]. Figure 2A illustrates the overall fold of the Ago2 mid domain model bound to the first 5' nucleotide of guide RNA. Conserved polar residues within the mid domain (Figure 1B, black highlights) contribute to this pocket, with the side chains (Y123, K127, Q137, and K163, numbered according to 1w9h) forming specific polar interactions with the 5' phosphate group (Figure 2A, black dotted lines). The aromatic ring of the Y123 side chain also stacks against the base of the 5' guide nucleotide. These residues are invariant among Ago family sequences and define the only conserved motif in the mid domain. Strikingly, the two identified MC motif aromatic residues (F470 and F505) reside over 20 Å from each other, making eIF4E-type stacking impossible (Figure 2B, magenta). Without this stacking interaction between aromatic residues, additional residues must contribute to any detected Ago2 cap binding.

The Ago2 structural models allow an inspection of the spatial surroundings of individual aromatic residues that were suggested to bind cap. The first identified Ago family MC motif residue (F470) points towards the hydrophobic core of the domain (Figure 2C). Residues that are near the F470 side chain either contribute to the hydrophobic core (L467, L474, V547, L563, and L560) or are neighboring on the helix (H466). The buried nature of the identified residue leaves little interaction surface for binding cap. Mutation of this phenylalanine to a smaller residue such as valine, for example in the double mutation evaluated by Kiriakidou et al. [1], would create a void in the core that likely decreases the stability of the mid domain fold. Measuring the activity of Ago2 endonuclease domain towards cleaving target RNA does not necessarily reflect the folding or structural properties of the adjacent mid domain. For example, a recombinant *Dm *Ago2 piwi fragment, which includes the RNAse H-like endonuclease domain but lacks most of the MC sequence region (including both aromatic residues) cleaves target RNA[18]. Thus in the background of a hydrophobic core F470V mutation, functional analysis of the mid domain becomes difficult to interpret.

The second identified aromatic side chain (F505) is surface exposed (Figure 2D). Neighboring residues that could potentially contribute to a cap-binding site (E502, R506, K509, and V129) are somewhat conserved among close metazoan Ago sequences: although E502 is replaced with G in chicken Ago3 and rabbit Ago2 and R506 represents only a conserved positive charge. Important functional binding sites typically retain higher levels of conservation. The E502 side chain resides one turn away from the F505 aromatic ring on the same face of the second mid domain helix. This placement might allow hydrogen bond formation between E502 and a guanine base stacked with F505. However, such a binding mode would require rearrangement of the modeled side chain orientations of these two residues. Similarly, the R506 and K509 side chains could contribute positive charges to binding cap phosphates but would also need to adopt different conformations.

Perhaps a more plausible cap-binding site would be located in the conserved Ago 5' guide RNA binding pocket (Figure 2E). Residues in this pocket are situated to bind phosphate at the 5' position of the ribose ring (Y123, K127, Q137, and K163) and form a base stacking interaction with nucleotide (Y123). The m^7^Gppp cap retains a similar 5' linked phosphate on the ribose (Figure 2F) that could mimic the 5' nucleotide from guide RNA. Interestingly, a phosphate from the third guide RNA nucleotide is positioned near the 5'phosphate, forming hydrogen bonds with another conserved Ago residue (N155). Each of these phosphates coordinate a divalent metal ion located in the binding pocket. The phosphates of the m^7^Gppp cap could adopt a similar conformation as the phosphate/divalent metal/phosphate in the Ago2 binding site. Unfortunately, such a binding mode would compete with guide RNA, suggesting that any detected cap binding to Ago2 is artificial.

### Structural context of reported Ago2/eIF4E alignment

**Figure 3:**
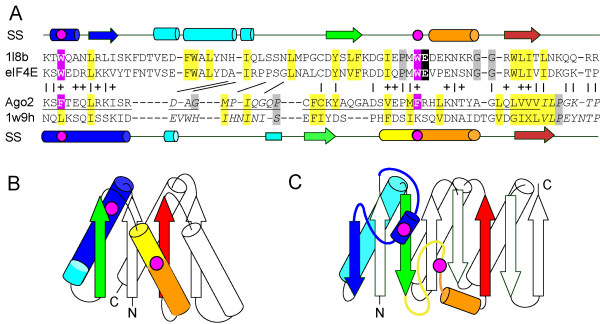
**Ago2 MC sequence region/eIF4E alignment and structure topologies**. (A) The reported [1] Ago2 MC sequence region alignment (Ago2) to eIF4E (1l8b) is depicted together with an aligned piwi structure sequence (1w9h) and with the BLAST-detected *Ce *eIF4E-1 sequence (eIF4E). The reported alignment (underlined) differs from the detected BLAST alignment in length and in placement of gaps (Ago2 residues that differ are italicized). BLAST identities and similarities are indicated between the sequences (with | and +, respectively). Differences in gapped regions are marked with slanted lines that pair BLAST residue matches. Sequences are highlighted according to family conservations as in Figure 1, with eIF4E cap stacking residues and the corresponding Ago2 aromatics highlighted magenta. Secondary structural elements derived from the respective structures (arrow for strand and cylinder for helix) are indicated above and below the sequences and colored by rainbow from N-terminus (blue) to C-terminus (red), with the positions of identified aromatic residues marked by a magenta circle. (B and C) Topology diagrams illustrate Ago mid domain (B) and eIF4E (C) connectivity. Secondary structural elements of each fold are colored as in (A). Magenta circles represent the relative positions of aromatic residues. The N-termini and C-termini are labeled.

None of the sequence detection methods used in this report identify a link between Ago and eIF4E sequences, even at very low confidence thresholds. However, the detected similarity between Ago2 and the eIF4E [1] is credible at first glance (Figure 3A). The reported MC sequence region alignment differs from our most confidently detected BLAST alignment in length and placement of gaps. The BLAST alignment positions one long gap in the Ago2 sequence (between the ISR and the DAG, slanted lines mark BLAST residue matches) that omits a core secondary structural element of eIF4E (Figure 3A, cyan helix), while gaps in the reported alignment interrupt secondary structure elements (Figure 3A, cyan helix and green strand). Such omissions and interruptions of core secondary structural elements cast doubt on the validity of the alignments between the Ago2 MC sequence region and the eIF4E motif.

The detected sequence similarity between Ago2 and eIF4E probably arises from chance localized structural similarity. Short stretches of sequence containing the identified aromatic residues anchor each side of the gapped region. The first aromatic residue resides in an Ago2 core helix (Figure 3B, blue) and in an eIF4E peripheral helical segment (Figure 3C, blue). This similar context is reflected in conserved local hydrophobicity profiles (Figure 3A), even though the secondary structure elements differ: an edge strand from eIF4e is aligned with the C-terminal portion of the first Ago2 helix (Figure 3, blue elements). The second aromatic residue falls within another core Ago2 helix (Figure 3B, yellow/orange) and N-terminal to an eIF4E peripheral helical segment (Figure 3C, orange). Although the local hydrophobicity profiles surrounding the second aromatic residue do not match, an unusually hydrophobic β-strand follows each (Figure 3, red). Despite these localized similarities, the overall fold topology differs between Ago2 and eIF4e, with the gapped region β-strand pointing in opposite directions in each respective fold.

## Conclusion

The mismatch and breaking of secondary structural elements, the topological differences that place the aromatic residues in different parts of the folds, and the marginal BLAST scores (best detected E-value 0.17) that disappear upon subsequent rounds of PSI-BLAST all question the validity of the MC sequence region alignment to eIF4E. Although localized sequence similarity can indicate common function, care must be taken to distinguish between sequence similarity arising from functional constraints within folds and similarity arising from chance matches in short sequence stretches. In the absence of confident similarity scores, assignment of common function to different sequence families requires additional evolutionary and structural justifications. The sequence grouping and alignment illustrated in figure 1 outline the evolutionary context of the Ago family, revealing the common sequence requirements for the structure and function of the mid domain fold. The multiple sequence alignment highlights a sequence motif in the mid domain that binds the 5' phosphate of guide RNA. Comparison of the structure of the MC sequence region in a homology-based Ago2 model to that of the cap-binding motif in eIF4E reveals some very localized structural similarities captured as statistically insignificant sequence similarities by BLAST.

Not surprisingly, the MC sequence aromatic residues are not homologous to the eIF4E tryptophans and are not positioned within the Ago2 structure to form stacking interactions with the cap base. The authors use a double Ago2 mutant (F450V and F505V) to support the role of these residues in cap binding. The F450V mutation alone could destabilize the mid domain structure enough to result in decreased detection of any measured binding. Assuming the surface exposed aromatic residue (F505) could stack with the cap base, additional surrounding residues should be required for binding in the absence of a second stacked aromatic residue. The surrounding residues do not display conservations typically observed for important functional sites. Alternatively, a more plausible binding site for mRNA cap would be in the 5' guide RNA binding pocket, given the similarities between the two ligands.

## Methods

### Sequence analysis

Collection of Ago family sequences and detection of homologous relationships between piwi domains were carried out with PSI-BLAST[19] searches against the NCBI NR database (Posted date: Apr 10, 2008; 2,979,120 sequences) or the SwissProt database (320,363 sequences). To confirm homology between the procaryotic piwi mid domain and the Ago family of sequences and to establish an initial alignment, a sequence corresponding to the mid domain of the *A. aeolicus *Argonaute structure ([PDB: 1yvu], range 349–484) was used as a PSI-BLAST query (E-value cutoff 0.005, maximum 20 rounds, NR database). E-values corresponding to initial detection of hits during the PSI-BLAST procedure are reported along with the round in which they are first identified and the sequence range of the resulting alignment. The initial and final coverage are calculated as the quotient of either the initial sequence range or the sequence range after convergence of the PSI-BLAST procedure and the length of the query (135 residues). As an attempt to recreate the reported BLAST link to eIF4E[1], a PSI-BLAST search using the same query against the reported database (SwissProt) was initiated, keeping hits below an extremely low confidence threshold (E value 100) for inspection.

To visualize the relationships between identified mid domain homologs, collected sequences were filtered to remove redundant sequences (cd-hit[20] cutoff 100%). All against all pair-wise sequence similarities were calculated using BLAST implementation (-pval 0.1) with the CLANS application[21]. Sequences were clustered in two-dimensional space with a P-value cutoff of (1E^-05^) until node movement became negligible (8804 rounds). Resulting clusters were colored according to visual groupings or according to species superkingdom for divergent or sparsely clustered sequences.

Ago and piwi-like sequences were aligned using the MAFFT server (version 6) [22,23] with default values (relatively accurate choice), and sequences were filtered for 100% redundancy using the Jalview alignment editor[24]. Secondary structures for the Ago2 sequence were predicted using the JPRED3 server[25]. The results from additional profile-profile based sequence detection methods (COMPASS[16] and HHpred[17]) were evaluated using the Ago2 mid domain sequence as a query (gi|29171734, range 445 to 565) against profiles built from PDB70 sequences. Structure templates were aligned using DaliLite[26], and the final multiple sequence alignment was assembled from the results of PSI-BLAST, COMPASS, and HHpred alignments, with some manual adjustments (mainly in loops) based on conserved hydrophobicity profiles and predicted or observed secondary structures.

### Structure Modeling

Structure models of the Ago2 mid domain (gi|29171734, range 445 to 565) were built using the alignment interface of SWISS-MODEL workspace[27]. Ten structure templates of piwi domains are available in the PDB. Each template corresponds to one of the three protein sequences depicted in Fig 2A: [PDB: 2f8s], [PDB: 2f8t], [PDB: 2nub], and [PDB: 1yvu] for *Aa *Ago; [PDB: 2bgg], [PDB: 1ytu], and [PDB: 1w9h] for *Af *Piwi, and [PDB: 1z25], [PDB: 1z26] and [PDB: 1u04] for *Pf *Ago. Input alignments of the Ago2 mid domain with most of the identified structure templates yielded models with similar final total energies (1ytu, -3418 KJ/mol; 2bgg, -4004 KJ/mol; 1w9h, -4050 KJ/mol; 2f8s, -3922 KJ/mol; 2f8t, -4303 KJ/mol; 2nub, -3847 KJ/mol; 1yvu, -3748 KJ/mol; and 1z25, -3420). The most favorable reported final total energy model (2f8t, -4303 KJ/mol) was generated from a template with a higher resolution (3.1A) and poor electron density for the second mid domain helix. Therefore, we chose the final models for illustration of the MC region based on a template with the best resolution (1w9h, 1.95 Å, -4050 KJ/mol) and a template with bound guide RNA (2bgg, 2.2 Å, -4004 KJ/mol), which represent *A. fulgidus *Piwi alone[8] or complexed with an siRNA Duplex[28], respectively.

## Competing interests

The authors declare that they have no competing interests.

## Authors' contributions

LNK and NVG designed the research, analyzed the data, and wrote the paper.

## Reviewers' comments

### Reviewer's report 1

Arcady Mushegian, Stowers Institute for Medical Research

The manuscript by Kinch and Grishin is an exhaustive refutation of an incorrect hypothesis which, however, led to some interesting observations (see below). The hypothesis by Kiriakidou et al. [1] is that the "MC motif" of human Argonaute2 and the eukaryotic translation factor eIF4A are related in sequence, presumably in structure, and in the mode of purported interaction with the mRNA cap structure. The refutation states that the "MC motif" is in fact the homolog of the known, structurally characterized Mid domain (or subdomain) of the PIWI proteins, which has structure different from that of eIF4A; that the residues in Ago2 implicated in interaction with the cap are in fact higly unlikely to do so; and that therefore the results of Kiriakidou et al. [1] need to be reevaluated.

The computational evidence presented by Kinch and Grishin is solid, and it not only shatters the computational "observation" of Kiriakidou et al. [1], but also holds extremely well against their wet-lab experiments. The fact that computer analysis, when done correctly, can give a stonger argument than biochemistry is not exactly news (see [29] for a brief discussion of the epistemological issues that are relevant here); what perhaps is more surprising is that the peer-review process in the high-profile journals like the one that published the work of Kiriakidou et al. apparently did not involve any of the state-of-the-art but freely available approaches utilized by Kinch and Grishin.

I have only two suggestions. First, even though Kiriakidou et al. most likely have misinterpreted their own site-directed mutagenesis results, their basic observations of cap-binding and other biochemical properties of the wild-type Ago2 may as well be real, and should not be (at least yet) thrown away – perhaps the authors could state not only what they refute, but also which parts of Kiriakidou et al. remain standing.

#### Author response 1

The basic observations of cap-binding and other biochemical properties of the wild-type Ago2 measured by Kiriakidou et al [1] may be real. However, these data and the resulting Ago2 cap-binding competition model of translation repression are debated in the miRNA community. Given the confusing and contradictory literature surrounding microRNA translation silencing, we hesitate to comment on the biochemical properties of Ago2 measured by Kiriakidou et al. [1] in this bioinformatics oriented report. Alternatively, we point to a review about microRNAs and translation where Kozak questions the validity of both the cap binding measurements and the translation inhibition studies from this paper, stating the authors' conclusion about tethered AGO2 translation inhibition of capped but not uncapped mRNA "is unwarranted" [30]. In a related miRNA system from Drosophila, Eulalio et al. perform experiments that seem to contradict the results of Kiriakidou et al [1], finding that an Ago1 double mutant (corresponding to the two identified aromatics) abolishes silencing without affecting the measured m7G cap binding. Instead, the double mutant eliminates Ago1 interaction with miRNA and with an argonaute hook protein GW182 [31].

Second, the manuscript should be refashioned as a discovery note, deleting a few words here and there, folding the content of the two tables into figure legends or Methods section, and making one composite out of the three figures – they are all parts of the one statement, i.e., "MC region is the conserved Mid domain of PIWI, distinct from eIF4A and unlikely to interact with the cap", and current Figure 1A is perhaps a distraction – after all, the point is that Mid domain is found in most clans in Ago/Piwi superfamily, not that these clans exist.

#### Author response 2

Normally, we would report such findings as a concise discovery note. Given the circumstances outlined below, we decided to keep the longer format of this article. We originally submitted a short Correspondence of our findings to the high profile journal that published the Kiriakidou et al paper[1]. The correspondence was rejected on the basis of lacking experimental evidence. The reviewers expressed a hesitation to believe structure models, with one referring to our work as speculative. These apprehensions are common among many scientists, partly driven by publications like Kiriakidou et al[1], where misapplication of powerful computational methods yields questionable hypotheses that lead to incorrect conclusions. Such responses could also arise from omitting too many experimental details and explanations from the very short format of the rejected communication.

We included quite detailed methodology and figures that may seem redundant in justifying the relationship between Ago2 and piwi in this report, especially for those accustomed to bioinformatics approaches, their strengths and their weaknesses. Without a certain detailed familiarity with the methods, sequence similarity search results are easy to misinterpret. Accordingly, many similar instances (to that of Kiriakidou et al) of flawed computational analyses have been published (see [29], suggested by reviewer 1 and Reviewer 3 below). Such studies obscure the power of computational methods and promote a general feeling in the research community that conclusions obtained through computational experiments do not constitute strong enough evidence. Our detailed descriptions might provide helpful guides to scientists working in the miRNA field, especially for those working on Ago proteins. Although convincing researchers with pre-formed opinions is particularly difficult, we think our description contains an educational component that could help developing minds.

We agree that Fig. 1A may seem distracting. However, illustrating the overall relationship between piwi-like sequences not only supports existing classifications, but also helps explain why the link between the mid domain of piwi structures and Ago sequences can be missed (and perhaps mistaken for having an incorrect motif). As a final comment, our original submission took over 6 months to be rejected, with the journal ultimately failing to fairly re-evaluate the published paper (Kiriakidou et al) [1] that was clearly based on questionable interpretations. See comments of Reviewer 3.

### Reviewer's report 2

Chris Ponting, Oxford University

Kinch & Grishin report a re-analysis of claims of Kiriakidou et al. [1] that a "motif within the Mid domain of Ago proteins bears significant similarity to the m7G cap-binding domain of eIF4E". This re-analysis brings together sequence and structure-based evidence in a statistically sound framework that does allow such claims to be evaluated appropriately.

The authors' re-analysis finds no evidence for statistically-significant sequence similarity between eIF4E and the Ago Mid domain; moreover, structural evidence provides strong evidence for these domains *not *being homologues. It is clear also that previous claims [1] that many Ago sequences lack a Mid domain are unfounded.

This paper goes beyond demonstrating the considerable value of sequence- and structure-based analyses. It also calls into question the interpretation of previous results [1], specifically stacking interactions between aromatic residues, typical of eIF4E, are incompatible with what is known of the Mid domain structure. The manuscript thus has considerable value in casting doubt on some of the conclusions drawn in a recent high-profile publication [1].

### Reviewer's report 3

Igor B. Jouline (Zhulin), University of Tennessee

In this paper, Kinch & Grishin, verified a previously published claim that a short sequence region in a human Argonaute protein (Ago2 MC) is similar to a motif in eIF4E that contributes to mRNA cap-binding [1]. Kinch & Grishin performed a carefully designed computational sequence/structure analysis, which led them to a conclusion that there is no similarity between the regions of Ago2 and the cap-binding protein. Thus, this study continues the trend of exposing erroneous sequence analyses that have led to questionable "discoveries" by experimental verification of computational predictions (see [29]). As recent history shows, it is very unlikely that a correction by computational scientists will force experimentalists who published original finding to go back, reexamine their work and admit "the wrong doing". However, it is critically important to unravel and expose such errors. Therefore, this paper is significant.

Overall computational approach taken by authors is both straightforward and state-of-the-art. They began by the most sensitive sequence-based search (PSI-BLAST) and followed through with careful multiple alignment and then structural modeling. The entire approach is described in a great detail, so it can be easily reproduced. I have absolutely no concerns with respect to how this study was designed, executed and presented.
